# The Vinnicombe Bequest: A Narrative of Hospital Work and Hospital Wants

**Published:** 1888-06-02

**Authors:** 


### The Vinnicombe Bequest: A Narrative of Hospital Work and Hospital Wants.

Upon a ledge in a long irregular slope of grass, which forms one of the lower declivities of a range of west country hills, purple with heather and decorated here and there with a patch of gorse, stands a house of moderate pretensions, just toning down from its first upstart brilliance under the somewhat blustering tuition of the wind and weather to which it is exposed, and growing more into harmony with its wild and beautiful surroundings.

Ten years ago, Mr. John Timberlake, at that time of Basinghall Street, in the City of London, wool merchant, came to the conclusion that he had worked long enough and made enough money—two rather difficult conclusions for a successful man of business to arrive at—and that he might now really set about carrying into effect a promise he had made to his wife ever so long ago.

### Mr. Timberlake's Promise.

Twenty years ago before it was, when they roamed together one holiday time over the western counties, and grew enthusiastic about their beautiful and varied landscapes. They were young people then—not overburdened with wealth, with their way to make in the world, and perhaps a little doubtful if it ever would be made.

So when the wife took her husband's hand into her own—they were sitting together upon a big granite boulder, overlooking a wide stretch of wild moor and wooded valley, with the blue sea beyond—and said: "I want you, dear John, to promise me that when you retire from business"—he was so startled by the abrupt reference to the far distant and almost impossible time that he answered at once with a gasp, "Yes, dear, I will," and then had to inquire what it was she meant to ask.

"I want you," continued she, quite seriously, and heedless of the interruption, "to promise me that you will try to buy this piece of ground and build a house on this very spot, and let us come to live here."

"My dear," said John Timberlake, "I will," and being somewhat recovered from the shock, he laughed gently, and by the help of a stone lying near by, he chipped off a small piece of the rock on which they sat and bid his wife keep it in token of the promise.

There was considerable reason for doubting at one time whether John Timberlake would ever have the opportunity to keep his word to his wife, for after showing signs of growing weakness for some months, alarming symptoms were suddenly developed, and it looked very much as though poor Mrs. Timberlake would never call upon her husband to redeem his promise. The family doctor gave her up, but he said there was just a chance, and advised a consultation with a surgeon who had suddenly acquired a great reputation by reason of his brilliant achievements in regard to cases hitherto deemed hopeless. The great man added one more to his successes when he took Mrs. Timberlake in hand, and in a few months she declared, with every evidence of reason, that she had never been better in her life.

### Mr. Vinnicombe Gives Trouble.

"My dear," said Mr. John Timberlake to his wife, who had followed him to his study one morning, and who stood looking thoughtfully through the window at the well-kept lawn, "this is a troublesome business of poor Vinnicombe's. Heaven knows I want to do what is right by my old friend, but I wish it had fallen to someone else's lot to carry out his wishes. I don't like the responsibility of dealing with all this money, and I am afraid I shall have to go up to London, and, altogether, if it didn't sound unkindly, I should say that I am very sorry he made me his executor."

"John!" returned his wife, with as much rebuke in her tone as was consistent with the sweetness of her disposition, "do think how great power of doing good he has put into your hands."

"I think," returned John, "that men should do good themselves, and not by deputy. Why did Vinnicombe leave it to me after he has gone out of the world, to do that which, if it's to be done at all, he ought to have done while he was in it? Besides, I am not altogether sure, my dear, I wholly approve of the instructions I must follow. I daresay hospitals are, on the whole, deserving places, but I've never had anything to do with them, and I *have* read things about them I don't altogether like. Somebody said the other day that they fertilise the field of pauperism, and for my part I should think it is not unlikely they do. Why, I'm told they positively dole out physic to all comers. And then there's this vivisection. Now, you as a woman—for women are supposed to be tender-hearted—ought not to approve of that."

### Dr. Rowe.

What Mrs. Timberlake would have answered is not known, for at this moment Dr. Rowe's gig, with the doctor driving, came smartly up the carriage-way, and the doctor having alighted with much agility, considering his bulk, was at the study door almost before Mr. Timberlake could make his way thither to receive him.

"Ah, Doctor! Prompt as ever. This is really very kind. Glad indeed to see you."

"How d'ye do?" said Dr. Rowe to Mrs. Timberlake. "Well it's pleasant to see you both about. I really didn't know what was the matter when I got your urgent message."

"It is not like my husband," said the lady apologetically as she passed out into the garden, "to put anyone to unnecessary trouble, but I really think he can't get on without you, and I do hope you will be able to help him."

"Exactly so," quoth Mr. Timberlake, dragging the doctor inside the study and shutting the door. "To speak truthfully, Rowe, there is a very great deal the matter, and I know no one else who can help me to put it right."

"Well, well," cried the Doctor cheerily; "I'm always at hand when I'm wanted—it's the patient who is not always forthcoming, you know, with some of us. But you are a little oracular this morning—who is it?"

"I suppose," said Mr. Timberlake, "this must be dubbed the patient"; and he held up a portentous legal document of several folios, marked here and there with bold marginal notes in pencil.

"Humph!" said Dr. Rowe, "voluminous notes of the case indeed! but you are not going to ask me to read them, I hope."

### Vinnicombe's Bequest.

"Not all," replied the other, "it's a purely local affection I want to draw your attention to; here it lies, Doctor," and he placed his forefinger upon a passage marked with a very bold cross indeed. "Listen—

'And I further give and bequeath to the said John Timberlake the sum of one hundred thousand pounds on trust, to apportion the same in such amounts as he may see fit among such hospitals for medical and surgical treatment, situate within the limits of the London postal district, as he in his uncontrolled discretion may select.'"

"Bravo!" cried Dr. Rowe, rubbing his hands vehemently.

"All the delights and honours of philanthropy thrust upon you at someone else's cost! Capital!"

"Vinnicombe was very fond of physic," said Mr. Timberlake reflectively, "no wonder his thoughts turned to hospitals; but if he'd only taken example by what's his name in the play, and left them the empty bottles, what a deal of trouble he would have saved me! Now seriously, Doctor, I am dreadfully concerned about this; what do I know about the hospitals within the limits of the London postal district, eh?"

"Not as much as you ought, I'll be bound, considering you lived there a quarter of a century; but you'll have ample opportunity now to atone for past neglect, I can tell you. Ha! ha! they won't let you rest in ignorance long. Enterprising secretaries will soon be on your trail, my dear friend."

"They've begun already," cried Mr. Timberlake with a tone of genuine anxiety, "and that's why I sent for you. The will was only proved last week, and I had thirty-seven letters with twenty-five book packets by yesterday's post, and there are fifteen more this morning.

"And very pretty reading, too, I can guess," said Dr. Rowe with a chuckle of delight. "Besides, what's the bother of receiving a few letters?"

"A few Letters!

"I've had scores!" groaned Mr. Timberlake.

"Yes, a few letters, compared with the intrusion of callers. Living down here you are saved that, at any rate. Why, my dear sir, if you were in London—aye, or within twenty miles of it, you would be regularly besieged. Do you remember the opening lines of Pope's Prologue to the Satires:—

'Shut, shut the door, good John! fatigued, I said;
Tie up the knocker, say I'm sick, I'm dead.'

They would express your feelings to a nicety then; but, as it is, you have quiet and leisure to think. Tell me, have you formed any definite plan yet—have you digested the applications?"

"Formed any definite plan! Digested the applications! My good Doctor, I should need to be a Moltke with the stomach of an alligator to have done that. Besides, I have not attempted anything until I saw you. As to these letters, they don't help me a bit; they are all alike. So far as I can make out, every hospital is on its last legs, and must go to the wall if I don't see my way to come to the rescue with a part of Vinnicombe's bequest. It is a good argument to begin with, but it loses a little by repetition. I think something must be radically wrong if hospitals are in straits like these. Why don't they keep within their incomes? It isn't honest."

"There is," said Dr. Rowe, "something very radically wrong, indeed; but not on the part of the hospitals. As to keeping within their incomes, why that is just what they wish they could do, but some of them can scarcely be said to have any."

"How do they exist, then?"

"As beggars, more's the pity. They live from hand to mouth, and often have to go in debt for the loaves they buy to feed the sufferers they shelter."

"That is, they run up bills and leave the public to pay them; and, speaking as an old business man, I say it's shameful. If they can't meet their liabilities, let them put up their shutters."

Death or Debt.

"What!" cried Dr. Rowe, with mingled indignation and amusement, "turn the poor people out of their beds into the street to die in the gutter? I tell you that is the alternative —die in the gutter! Remember, not a bit of provision for treating them is made by the nation or the Government. The whole of this duty is left to the voluntary hospitals, and when private citizens are found willing to discharge this great imperial duty at their own cost, the only way the Government can find to show its appreciation of their services is to tax them."

"Impossible!" cried Mr. Timberlake.

"Only the other day an attempt was made to enforce the tax on man-servants in the case of the porters and male nurses of a hospital, and though it may not be usual to go to the length of that absurdity, yet the fact remains that the London hospitals are made to pay thousands a-year towards poor and other local rates. Besides, as to leaving the bills to be paid by the public, that is exactly what ought to be done; and if the public had any conscience in the matter, every individual would consider it a privilege to pay his share of a common debt. Now take yourself, Timberlake; look at the debt you owe to hospitals ——"

"I?" remonstrated Mr. Timberlake. "Really ——"

"I'm not going to ask you how you've discharged it," continued Dr. Rowe; "but look at the debt itself."

What have the Hospitals done for Me?

"My dear boy, what have the hospitals done for me?"

"They brought your wife out of the jaws of death," said Dr. Rowe impressively, "and gave her back to you strong and well."

"Surely you are dreaming. Neither my wife nor I eve entered the door of a hospital that I know of."

"I quite believe that," returned the Doctor; "but the surgeon who saved her entered it a great many times, and brought away all the knowledge and skill he possessed from the work he had seen carried on within those walls. Speaking humanly, it is as certain as anything can be that it is owing to the labours of our hospitals that your home, for one, is not desolate."

Mr. Timberlake grew thoughtful, but did not yield to this argument without a struggle, though in the end he admitted there was something in it; and Dr. Rowe, making the most of the concession, laid down the position with much emphasis and some fervour.

"So," said he, summing up, "it comes to this—if there were no hospitals there would be no knowledge of medicine and surgery, and all the gifts which science is ready to put into our hands would be lost for want of the power to turn them to account."

Mr. Timberlake, whose acquaintance with hospitals was chiefly gained from certain cynical criticisms which he had read from time to time, and whose old mercantile spirit was especially impatient of their impecuniosity, was perhaps one of the very last persons in the world to whom the task of distributing a fund for their relief should have been entrusted. But at least he had this recommendation—he was conscientiously desirous to do his duty; and it was therefore with great satisfaction that he found his friend Dr. Rowe, in whose judgment he confided, was deeply interested in the matter under consideration, and ready to help him in every way he could.

Friends in Council.

So, the Doctor's mare having been put into the stable, the two sat down to pass the morning in conference.

"As he in his uncontrolled discretion may select," repeated the Doctor. "Well! there is no curtailment of liberty there. Are there any complications about the estate—any realty, or what the lawyers call impure personalty?"

"No difficulty is likely to arise. All in perfect order—scarcely any debts, no real property, legacies few, all investments in first-class Government securities, which can be realised at an hour's notice."

"Capital! I think you are a man to be envied. Why, you will be one of the biggest lions in the hospital world. They'll fête you—you will be mobbed—you will have to be presented at Court."

"Gracious goodness, don't say anything like that to the wife, Rowe."

"You will be made a Baronet—Sir John and my Lady Timberlake."

"Ah, very pretty indeed," laughed the other; "and the record of services for which the honour was granted—giving away what didn't belong to him."

"Precisely; an act which constitutes an enormous claim to consideration. But, anyhow, you will have to go to London; and, put shortly, what I would advise you to do now is to settle a provisional list of the hospitals you intend to help. Then go and see them and judge for yourself, and modify accordingly."

"That is what I should like to do. But just one question: Will you see me through with this? will you come with me?"

"Certainly I will. I never enjoyed the prospect of anything more in my life."

"Well then, to business. I haven't been altogether idle. I sent for this list of London charities, and there are those fifty-two letters. How many hospitals are there in London?"

"Upwards of ninety, if we accept their own definition in all cases," replied Dr. Rowe. "Half of them, perhaps, are rather worse than useless. However, there will be no lack of those which are undeniably good, so we need not sit in judgment on the rest."

"Can you explain," asked Mr. Timberlake, "why it is that while so many forms of charity flourish, the hospitals languish? Do they command the full confidence of the public?"

"Depend upon it," answered Dr. Rowe, "if they did not the numbers of patients would soon fall away. The question of confidence does not appear to arise. I am disposed to think that thoughtlessness, and that curious meanness, which delights in getting something without giving anything in return, explain everything."

"I always supposed that hospitals were held aloft as good examples of the nation's munificence. Are they not so?"

### What does the Nation do?

"If by the nation you mean a few self-sacrificing people, who take upon themselves to discharge one of the first duties of the community, yes; but as to the nation itself, a hundred times no. Do you know that the only endowed hospitals we have owe their endowments to past generations, and that of the present barely ten people in a thousand give anything whatever?"

"You astonish me."

"Most people are astonished when they hear that for the first time. Yet it has been repeated often enough."

"Is any class neglectful more than another?"

"I think not; they may all be impeached alike. Charity generally owes a great deal to the active co-operation of the members of the most exalted family of all, and if a fair part of the nation followed their lead hospitals would have little to fear; but, nevertheless, such work as hospitals perform is right royal in itself, and ought to need no advocacy."

### Do Hospitals Pauperise the Patients?

"Is it not a charge against hospitals that they encourage pauperism and demoralise their patients?"

"In individual cases they may be taken advantage of, but as a general statement the accusation is singularly inaccurate. Rather say their work effectually diminishes pauperism. A sick workman, if he cannot be cured must become a pauper. The hospital puts him on his legs again, and enables him to resume work and to support his family. Think what the nation would be without the hospitals. It would be a nation of paupers. The most that can be said is that the classes which are directly relieved by hospitals share the apathy of those socially above them, concerning the ways and means of their benefactors. We talk about the demoralisation of the poor because they neglect to give something of their poverty; but what is their offence compared with that of the multitude of people out of reach of the pinch of poverty who, when they want to get their poor relations, their servants, or their other nominees into the hospitals, will put themselves to any trouble and solicit favours from any friend or stranger in order to evade the payment of a trifling subscription? Ask the experience of any hospital as to this. Ladies of fashion will drive up to the door in magnificent equipages and sue for a free order, and when they have got it they will pose as helpers of the poor, accept all the gratitude offered them, and grumble at the demand to pay their nominee's washing bill. Tell me in which class is the demoralisation most complete?"

"Go on, Doctor," cried Mr. Timberlake. "I am learning something this morning."

"What is there to add," returned Dr. Rowe, "unless to say that, so far from the hospitals being testimonies to the nation's charity and benevolence, they are but monuments to the beneficence of a few individuals, and defaced by inscriptions of the nation's ingratitude and neglect? It is a black picture I will admit, but, thank heaven, it is relieved by many a ray of Divine light. No wonder I feel disposed to speak with some warmth about your present opportunity, for I know how valuable will be the help; yet we don't need a huge sum to arouse our feelings. I think that, notwithstanding the munificence of old Vinnicombe, I should give a higher place on the roll of honour to the little nursery governess who out of her earnings of sixteen pounds a-year saved five pounds, and brought them as a "thankoffering" to the hospital in which her only sister had lain and died of hopeless malady two years before. Aye, it is in the sunshine of such acts that our faith in human nature—the faith we all have some time in our lives—seems to lift its head and blossom upon the grave in which we thought it lay dead."

### The Voluntary System.

"It has occurred to me sometimes as a business man," said Mr. Timberlake—"as a business man," he repeated, as though he were entering his protest in that capacity against anything like poetical treatment of the subject—"it has occurred to me that institutions of acknowledged value ought not to be left dependent for existence upon chance contributions. What think you about that?"

"In regard to hospitals, I am disposed to agree, for the reason that a really imperial and indispensable work is hindered, and rendered less perfect than it might be, for lack of the means to carry it on. But the subject teems with difficulties, and nothing could be more disastrous than to place hospitals upon a parochial level. At present they are served by the most eminent members of the profession; and to be upon the staff of a great hospital is not only a coveted honour, but it is absolutely necessary to the attainment of the highest professional distinctions. Men such as are now attached to hospitals would never consent to work under a board of guardians. Again, if they did, the parochial restraint would effectually cripple their intellectual strength; and they would become as perfunctory in the discharge of their duties as they are now careful and laborious. Whether the hospitals could be maintained and controlled by the State without a similar deterioration in the quality of their work is more open to question. Perhaps under capable and sympathetic direction their scientific reputation would be preserved, but with the loss of that philanthropic and religious element in their management to which, as a medical man, I am glad to render my tribute of admiration."

"Then there is nothing to be done but to go on as before

### Hospital Sunday.

"I do not quite say that. The encouraging efforts made to awaken public interest may yet bear fruit. I should be disposed to attribute very great value to the Hospital Sunday movement, if only the clergy would bring their hearts into the pulpits with them. The notion of all the religious bodies combining, with the Established Church at their head, to promote a common fund in aid of the great national work of healing the sick is a grand one; and if the result is disappointing, it is because the clergy fail to use the advantages they undoubtedly possess. When I was in London, I heard many Hospital Fund sermons, but there seldom seemed any comprehensive grasp of the subject, which would so readily lend itself to graphic description and powerful pleading. Sometimes the only allusion was a bare announcement that a collection would be made in behalf of the fund. We want the sympathies of the congregations aroused, and when that was once accomplished there would be a better response than the sixpence per head which we are now told is the average contribution."

"But assuming," said Mr. Timberlake, "that the public concern is not to be aroused, and it seems to me that these repeated appeals—we must not forget my fifty-two letters, by the way—cannot fail to blunt the feelings of those who receive them, is there nothing between maintenance by donation and the revolutionary possibilities we have been discussing?"

### A Word to Life Insurance Companies.

"Well, of course, there is the old threadbare proposition of payments by patients, and no doubt something might be done in that direction. But as the patients will never be able to do everything we must look elsewhere too. I confess to liking the idea of obtaining the co-operation of the life assurance companies, or perhaps it would be better to say of those whose lives are assured. It is certain that but for the work of the hospitals the premiums payable would have to be enormously increased. Well, the premiums paid in London annually amount to twenty millions sterling, and it is a simple calculation that half-a-crown added to each £10 of premium and collected by the companies for the benefit of the hospitals would supply no less than £250,000 in aid, or enough for all their needs over and above their assured incomes.

"You mean," said Mr. Timberlake, reflectively, "that if I were paying a premium of £20, the company would politely ask me to add five shillings for the hospitals, to whose work I owe the fact that it is not £40 at least."

"Precisely," said Dr. Rowe, "and would you refuse?"

"I will answer your question by another—who would?"

"Well, if one half the insurers did, there would still be ample to solve the hospital difficulty for ever; yet," said Dr. Rowe, "the suggestion has been pooh-poohed by the wiseacres. I should like, however, to add this—that if a method of the kind should be ultimately adopted, the cause of religion will have lost one opportunity more to make its power felt. But the morning is going, and we must get on. Ah! thank you; that guide will be useful, and also this—the supplement to The Hospital, issued last year in connexion with the Sunday Fund appeal. The latter shows at a glance all the different institutions, their character and the amount of work performed."

### A Blot on the System.

"First we have the general hospitals, mostly of magnitude, and almost above criticism. Then comes the crowd of 'specials.' Very questionable places some of them, I am afraid—big-voiced in the matter of appeals, and many with sonorous titles which are little justified by their work. It is one of the greatest blots upon the voluntary system that anyone, however incapable, is at liberty to open a hospital, however unnecessary, and to appeal to the public to support the useless institution he has brought into being."

"Why is not this liberty curtailed?"

"There seems no means of curtailing it. I must not begin to sermonise again, but I will just remark that a great part of the impecuniosity of hospitals may be attributed to the depredations and importunities of worthless institutions. So we must be careful."

"What would you advise? Would you apportion the fund among a select few, or let all worthy institutions share?"

"If I were dealing with my own money I should certainly prefer to give the few substantial aid; but in distributing somebody else's I think you ought not to make choice, which appears invidious. On the other hand, I have often grieved to see magnificent funds dissipated in infinitesimal amounts among a lot of charities. Spread over too large a surface, the showers, which might fertilise, evaporate as quickly as they fall, and leave no field the richer."

"Let us try and avoid both extremes; and now for these letters. I've been thinking," said Mr. Timberlake, "what hungry lives these hospital managers must lead."

"Yes," returned Dr. Rowe, "they are always on the look-out for something to turn up. It isn't often they scent spoi like this, and they will make the most of the chance, as they ought."

When the two friends had sorted out the applications—Dr. Rowe insisting that the general hospitals should be put in a first category and the specials in a second, the latter with several subdivisions—they proceeded to consider the appeals *seriatim*.

### Which Should Get It?

Dr. Rowe's knowledge of the merits or demerits of the several institutions was tolerably complete, and his companion was well content, in most instances, to accept the advice offered without much question, and a goodly list of hospitals chosen provisionally to participate, as a lawyer would say, was soon figuring upon the sheet of foolscap paper where Mr. Timberlake, with great care and consummate neatness, had entered the name and such other particulars as he thought desirable for reference. There were large general hospitals and small general hospitals, hospitals for consumption, for sick children, ophthalmic hospitals, hospitals for fevers, hospitals for mental disease, and there would have been hospitals for every conceivable ailment if the applications had not been judiciously sifted under Dr. Rowe's guidance.

"Why," cried the latter, "one would think it needed as many hands to cure a patient as it takes, we are told, to turn out a pin. Stuff and nonsense, sir! This minute subdivision of medicine and surgery into a multitude of specialities is ruinous to the best work, and both science and patients suffer when the physician or surgeon sees nothing beyond the limited field of vision upon which his whole professional attention is riveted. At the same time, the area of knowledge is so vast now, and the problems of our later day life are become so complex, that no man's sight can penetrate everywhere alike, and there are, and it is well there should be, specialists of the right type—that is, whose specialism is not the root and branch of their knowledge, but its blossom and fruit. If I wanted to be dogmatic I should say that special hospitals are only wanted for maladies not readily treated in general hospitals, and that the only right kind of special hospitals for treating such maladies are those which possess the services of men who can bring to bear the microscopic powers of a single eye when it is necessary so to adjust it, but are equally capable of using both eyes and can sweep the whole landscape."

"I notice," said Mr. Timberlake, "that many general hospitals provide special departments for particular groups of diseases."

"Yes, it is so. They are becoming wise; but we must not forget that although the general hospitals are rightly more comprehensive than of old, there are still classes of diseases which need special accommodation."

"What next?" laughed Mr. Timberlake, almost interrupting his friend as he spoke, and waving a letter towards him, "Here is somebody writing about a hospital for nervous diseases. Ha! ha! that is rather amusing: a place, I suppose, for ladies whose nerves are weak, poor dears, and who need a tonic and a little change. I think we may put that on one side."

### What are Nervous Diseases?

"Oh! stop a moment," cried Dr. Rowe; "do not let us be over hasty. One of the strongest men I ever met with suffered from a form of nervous disease—aye, and it killed him, too."

"Doctor!"

"He was a giant of a fellow—a sergeant in the Guards, stood six feet two inches and measured I forget how many inches round the chest. A handsome face he had too. The surgeon of the regiment was a friend of mine. He was as proud of the men as if he had been the colonel, and always had a kind word for this sergeant. He almost cried when he told me what had happened. The poor fellow had gone into the hospital in Rochester Row then, and I went to see him. He lay like a log on the bed—his face drawn to one side, and every feature distorted. He had been unconscious for the first day or two, but he had roused a bit. He was unable to say a word, but he had the use of his right hand, and he wrote with a pencil on a slate: 'It's only something the matter with my nerves.' Forty-eight hours after he was dead."

"Goodness gracious!" cried Mr. Timberlake; "what was it?"

"An effusion of blood on the brain—the seat of our nervous organisation. Yes, nervous disease embraces multitudes of the very gravest maladies humanity suffers, and includes alike the painless extinction of a living death and the racking tortures of agony indescribable."

"I never knew ——" began Mr. Timberlake.

"My dear friend, your ignorance is shared by an enormous number of usually well-informed persons, who little reck troubles from which they are happily exempt. It is as difficult for some unimaginative minds to comprehend sufferings familiar in the every-day experience of less favoured fellow-creatures as it is for a man who has been always rich to realise the cravings of want."

"My dear doctor, I am growing altogether ashamed of myself. A pretty commentary this is upon the powers bequeathed me. I really think as an act of penitence I ought to give the whole sum to the mitigation of nervous diseases—what do you say to that?"

"No," responded Dr. Rowe, "that would not be right, according to my way of thinking; but I should like you to visit the chief hospital where maladies of the nervous system are treated, and then if you are satisfied as to its utility, allow it to share in old Vinnicombe's bounty. Let me ask you always to bear in mind that whatever the value or importance of an individual hospital—and managers may well be pardoned for the *esprit* in regard to their own institutions, which carries them through so many anxieties and braces them to so great exertions—we ought to remember that the vast work carried on by the hospitals of the metropolis must be regarded as a whole ere we can form any definite opinion of the depth of its philanthropy or learn to appreciate the splendour of its achievements."

### Ask if You would Receive.

The next morning brought Mr. Timberlake a further supply of lugubrious letters from different hospitals which had not previously appealed, and several commendatory communications concerning some which had. Old friends came to light in the most unexpected way, and curiously enough every one of them was "much interested" in a particular institution, and having heard of the fund, "hoped," "begged," or "trusted," etc., etc.

A considerable number of these correspondents were ladies, and not a few wrote letters almost irresistible, while one or two forwarded photographs fascinating enough, one would have thought, to charm every sovereign of the fund to travel their way as obediently as the rats followed the pied piper; but Mr. Timberlake was a little impatient of these overtures, and the only effect was to render him somewhat irritable.

"Yes," said Dr. Rowe, "I can quite feel for you, my dear friend, but I feel for the advocates too. It is very easy to grumble at a man for asking, but remember, if you don't ask it is very little you get. How much do you think hospitals would receive if they stopped sending out appeals? Of course it's a pity they must, because of the expense, but there's little spontaneity about almsgiving, though, thank heaven! there's many a man who wouldn't think to give if he were not applied to, who gives liberally enough when the appeal is made. Some years ago I noticed that a very big and a very poor hospital got less than £600 in donations during the year, while another doing just a twenty-fifth part of its work got more than £3,000. Managers have learnt the lesson taught by this kind of thing, and unfortunately it has also come home to them that only a very limited class gives anything. So those who already give are deluged with appeals to give more, and those who never give are left alone.

I often wonder that one or another of the millionaires of London does not take up with some needy hospital and set it on its legs. It would not be beyond the strength of some of the great ground landlords for instance, and the subject would not be an uncomfortable one for reflecting upon when they themselves lay sick. There is too much frittering of great opportunities. What we want is a few men who would do something grand and heroic, and therefore lasting. They need not found new hospitals; they could do better than that—strengthen and render sure the work of those already in existence."

### Knowledge Brings Respect.

After a fortnight of careful consideration, aided by the reading of reports, books of reference, and frequent conversations with his neighbour, Mr. Timberlake had got a very fair idea of the hospital question, and had acquired a far greater respect for the institutions than he had before boasted. He was no longer inclined to be censorious; but, having for the first time in his life obtained a real insight into the great work they performed, he very frankly admitted that he had changed his opinions, and was becoming enthusiastic enough about his task to satisfy even Dr. Rowe. The more he learnt the less he found to grumble at, and the ancient bogies, which were but the offspring of darkness and ignorance, evaporated as thoroughly as a child's nightmare in the rays of the rising sun.

But for the little depression his feelings always underwent when he parted from his home, Mr. Timberlake was as happy and anticipative as man could well be when he stood on the gravel drive one fine morning in early June equipped for his journey to London. He patted the sturdy roan cob, which consorted so well with the substantial dogcart of those hilly parts, chatted cheerily with the groom, and positively beamed as he kissed his wife goodbye.

As they proceeded they came in sight of a small farm lying deep down under the shelter of a big brown hill, among a few enclosures redeemed from the wilderness round about. Bright and green they looked, for the hay-time on these uplands is long deferred, and the grass was not yet in flower, but waved young and tender. The farm, which stood miles away from any other habitation, consisted of a few scattered outbuildings of rough stone and a white homestead. The place had an untidy, neglected look about it, which grew more obvious as it was approached; and the beauty which seemed to lie round about when viewed from the distance melted away, and even the folds of the many-shaped hills disappeared and carried the whole landscape with them.

A Case for the Hospital.

"Have you heard how poor Russell is, Tom?" asked Mr. Timberlake of his groom.

"Not since the week afore last, sir; he was very bad then."

"Ah! we can spare a minute, just jump down and ask about him."

Tom returned with a gaunt, trouble-stricken woman, who had two children dragging at her gown.

"They took him away a fortnight agone," said she, wiping her eyes. "You see, sir, it was this way; we couldn't do what was right for him here, and Dr. Pinder of Northhoe said he must go into a hospital, and that there was only one place where they would take him, and that was in London."

"London!" cried Mr. Timberlake; "what's the address?"

But Mrs. Russell was not able to answer, for it appeared all had been arranged in a hurry, and she did not know anything about London, and she was "very upset-like," but she hoped they would cure him, and they would send a message to Northhoe, and then the doctor would come and tell her about it.

So all Mr. Timberlake could learn was that out of that wild upland solitude poor Farmer Russell had been given over in implicit faith to the keeping of a hospital in London.

When they drove up to the little station at Northhoe Dr. Rowe had just arrived, and by the time they had shaken hands and taken their tickets the train hove in sight.

A railway run of five hours brought the two friends to Reading, where they changed into a slow train, for Mr. Timberlake had arranged they would dine and sleep at his wife's sister's little place, and this was best reached from one of the stations a few miles west of Paddington. He had a distinct purpose in view when he accepted the invitation of his sister-in-law, for he knew that a great part of her quiet life was spent in ministrations among the sick, and he thought it would be very pleasant to have a chat with her over the business which had brought him to town.

Immediately the fly turned out of the dusty high road into the shady carriage-way leading to Fairlawn, the pretty little house owned and occupied by Miss Kate Verity, a sense of peace seemed to make itself felt. Everybody who visited the place was conscious of its presence, yet nobody was able quite to explain whence it came. It might have been merely the effect of the contrast between the broad, noisy highway and the leafy little avenue, where the only sounds besides the grating of the wheels upon the gravel were the soft rustling of leaves and the trill of birds in the branches of chestnut and elm and copper beech overhead. When they turned an abrupt bend in the drive the cottage was revealed, clad in leafage and blossom, with a bright awning stretching forward from the porch, and under it the lithe form of Miss Kate herself; one of those women still sweet and beautiful in the autumn of their lives, of whom we never cease to wonder that they are old maids, yet would not have them anything else, lest change should mar the perfection we worship.

Miss Kate Verity.

Miss Kate Verity was a lady whose modest fortune was employed in giving practical effect to her permanent conviction that all wealth is held in trust for the promotion of God's work; and when she had made provision for one or two needy relations, she felt at liberty to pursue her own plans in her own way. A considerable part of her income was given directly to various charitable agencies; another portion was dispersed unostentatiously among the necessitous in the neighbourhood, and, not content with this, she maintained about her, under the pious pretence that they were of use, several old friends who had fallen upon evil days, and shared the comforts of her home with a changing company of three convalescent women from different London hospitals. She was delighted to receive her visitors, and became enthusiastic about the bequest.

"I hope," she said, "you will not think that I am wanting in sympathy for all the other grand organisations devoted to religious and material purposes; but poverty always appeals to me irresistibly, and when I think of the poverty of our hospitals I feel not sorrow only, but shame."

"You," said Dr. Rowe, "have seen a great deal of hospitals, I understand, and from the best point of view to form an impartial opinion. Tell me if you are satisfied with the philanthropic side of their work."

"I cannot imagine," said Miss Verity thoughtfully, yet without hesitation, "more perfect devotion to work than I have seen in the wards of our hospitals. I cannot picture to myself a tenderer care than I have seen bestowed upon the sufferers, and I am able also to bear some testimony to the skill with which poor people I have known in their everyday life have been restored from sickness to health and usefulness."

The Fairlawn Establishment.

After dinner Miss Verity accompanied the two gentlemen through her little establishment. Mr. Timberlake noted many improvements and modifications since his last visit, and Dr. Rowe, to whom the whole was new, expressed himself in cordial terms.

They saw the rooms given up to the convalescents, and the three poor, pale women who had just taken the places of others who had gone back to their homes strong and well; they saw other women sitting by an open window plying their needles upon some bright, comfortable material which was being made up into jackets for hospital patients to wear while sitting up in bed; they looked in at the large apartment—once a billiard-room—where Miss Verity had her reading and working parties, mothers' meetings, and the like, and took a rapid survey of the kitchen, where sundry preparations were in progress for the weekly distribution on the morrow.

Then they descended to the garden—a maze of sunflecked trees, golden-leaved, with bay and pine and cedars silver-tipped, and winding in and out went serpentine walks flanked by broad bands of verdant turf with margins of rich colour, which surged like a surf of flowers right up to the lower branches of the shrubs.

There were two thatched arbours, draped in roses and honeysuckles all aglow with bloom, and clematis which promised a purple glory later on. Here, from time to time on summer afternoons, came poor neighbours to drink tea with the convalescents, and about the paths feeble children from close alleys in the adjacent town rambled and looked in speechless joy upon the wonders of the garden.

"And are you satisfied," asked Mr. Timberlake, "with the results of all your philanthropy?"

"Do you call it philanthropy to please oneself? I am not only satisfied; I am thankful."

"But of your protegés—do you find them grateful?"

"I do not look for gratitude—that implies obligation. I am content to gain their affection."

Flowers for the Wards.

"This," said Miss Verity to Dr. Rowe, leading the way under a rustic arch, "this is my own especial enclosure—it is my hospital garden. Every plant which does not yield blossom or foliage fitted for cutting and placing in a hospital ward is excluded. Twice a-week, as long as the flowers last—and I have reached now to nine months out of the twelve—I send up baskets to the different hospitals. It is a hobby of mine, and I receive so many cordial letters that I am sure my little labours are appreciated. As much as possible I tend these plants myself; it is a pleasant recreation for me, and my poor friends find such delight in cutting and packing the flowers for the fellow-sufferers they have left behind."

"I should like to know the secret of your system," said Mr. Timberlake; "everything looks as luxuriant as ever."

"Ah!" replied Miss Kate, laughing, "it is astonishing how everything seems to thrive. I think it is the love we bear them that helps us to tend them aright. A good nurse must have love and compassion you see; it is not only skill which counts. We have quite fun over this corner of my garden. Whenever we see a poor drooping plant we say, 'Come, you shall be admitted to the hospital,' and then we bring it in here, and it seems to grow strong again."

"What do you aim at most," asked Dr. Rowe; "colour or fragrance, or what?"

"I think that we must admit that our first requirement is the somewhat vulgar one—size. Durability is also very important. Large flowers are necessary for large wards These great red and white lilies are grand to look at; so are the delphiniums, and foxgloves, and zinnias: one spray lights up a room. Later on we get sunflowers and hollyhocks, and after them dahlias. But there is really an endless succession."

"I must congratulate you upon your success, Miss Verity," said Dr. Rowe, after a critical examination of the borders. "I know something of the worries and disappointments of gardening under our English skies, and I never saw plants in greater perfection than yours appear to be. I can readily understand how great a harvest of pleasure this plot must yield."

"Yes," said Miss Verity, "only marred by one drawback."

"Ah!" cried Mr. Timberlake.

"People will not return the baskets, John," said his sister-in-law.

### WITHIN THE HOSPITAL DOORS.

The next morning, after bidding adieu to Fairlawn, Mr. Timberlake and Dr. Rowe went early to London, intending to call upon the solicitor who was acting for the former in the Vinnicombe business, and having been assured by that gentleman that there was nothing to prevent the realisation of the estate within a few weeks, Mr. Timberlake gave himself up to the task of visiting, in company with Dr. Rowe, the different representative hospitals.

They went in a very unassuming way—Dr. Rowe merely handing in his card as a medical practitioner from the country—and so they saw the several institutions in their ordinary work-a-day condition. Not a soul knew that one of these gentlemen had a hundred thousand pounds to give away, and they obtained neither more nor less attention than other callers.

At no single institution was any difficulty made about giving them all the information they wanted, and it was noticeable that if only they got safely by that terrible personage, the hall porter, all was smooth sailing.

Mr. Timberlake, whose knowledge of hospitals was limited, as we know, to their external architecture—a feature far from universally prepossessing—was gratified beyond measure at what he witnessed within. He had shared the vague dread with which so many people regard the interiors of these institutions, and having pictured to himself a series of gloomy apartments tenanted by miserable sufferers, about whose couches ghoul-like attendants hovered, he would have shrunk from the ordeal of visiting them, but for the persuasion of his friend and his own conscientious desire to act honestly by the trust imposed upon him.

What was his astonishment to find not only that the proprieties of life were observed, but that many refinements and little elegancies were not neglected. Just as there was an almost utter absence from the wards of the unpleasant characteristics which usually mark the material atmosphere of a sick chamber, so there was a pervading sweetness of tone which seemed wholly in harmony with the legend of their birth. In place of the dull grey light and repulsive imagery with which the inexperienced are prone to invest a hospital ward, he found well-appointed chambers suffused with colour, decked with flowers, and vying with each other in homeliness and comfort. Contrary to the expectation that a hospital ward would contain the dregs of life's bitterest draughts, he found existence almost purged of its grossness. In the fiery furnace of affliction much of the dross which had encumbered the better nature had melted away, and not a little fine gold was laid bare. Suffering there was—terrible suffering—but it was often endured with a patience which rendered it sublime, and made as great demand upon admiration as upon pity. Even in the wards for children scarcely a whimper was heard, while in those for adults, once rough men and women lay chastened, yielding themselves ungrudgingly to the better influences and teaching about them.

It was as though, softened in hours of pain, their characters had taken the impress of the nobler promptings to which they were subject, and Mr. Timberlake, when he came to think over the matter, had little difficulty in sharing the belief that the hospitals are powerful auxiliaries of religion, and are carrying on a great regenerative work, for which the world at large, and especially that part of it which is bigoted in its philanthropy, gives them scant credit.

### LOOKING FOR RUSSELL.

In every hospital he had entered Mr. Timberlake had made inquiries in the hope of hearing something of his neighbour, Russell, but hitherto without success. One more afternoon remained available, and at Dr. Rowe's wish, this was to be devoted to visiting that particular institution which at an earlier period of his education had aroused Mr. Timberlake's impatience, and about which he still remained doubtful—viz., the hospital for the treatment of nervous diseases. On the morrow he proposed to search out some survivors of his old City friends, and the next day being Hospital Sunday, as the posters displayed about every hospital he had visited had afforded him abundant opportunities to learn, and none to forget, he proposed to wind up his visit in appropriate fashion by attending service, and to start for home the following morning.

### THE HOSPITAL FOR NERVOUS DISEASES.

The work of the hospital which had been placed last upon Mr. Timberlake's visiting list differed greatly from all he had seen already. Reckoned up in mere heads of patients it fell far short of that performed by the largest general hospitals, but among special hospitals this was one of the greatest, as the number of its beds approached two hundred. Moreover, it was remarkable for the uniform gravity of the sufferers' condition. Here were no trivial cases for which a single attendance might suffice. In nearly all, alas! were the germs at least of deep-seated and, if neglected, death-dealing maladies.

The building was a new one, and stood in an old-world neighbourhood, whence the tide of fashionable life had ebbed a hundred years ago never more to flow, leaving a peaceful sleepy emptiness, as if the last century, tired of its dissipations and late hours, had gone home to bed and was not yet awake again.

Turning out of a busy thoroughfare, the cab which carried Mr. Timberlake and Dr. Rowe travelled the length of two narrow and rather melancholy streets, then passed into a small but well-timbered square, and drew up before a building which, as Mr. Timberlake remarked with unconscious irony, did not in the least convey the idea of a hospital, as it was both bright and handsome.

True to his habit of thought, Mr. Timberlake was for finding fault with the managers on the score of extravagance, but Dr. Rowe reminded him that it need cost no more to make a building pretty than to make it ugly, and that it would be a pity, and indeed almost incredible, that any hospital should be built now without some external evidence of the more humane sentiments of the age.

Why Hospitals should be Attractive.

The first object of a hospital, said he, is to make sick people well, and that is not rendered an easier task by surrounding them with gloomy associations. It is astonishing how quick a sick man's eyes can be, and how his heart will fail him when he sees he is entering some dingy, dark edifice, which perhaps he has learned to shudder at even when he has passed it in health.

And if a measure of beauty and cheerfulness be desirable in all cases, it is pre-eminently so when we are dealing with sufferers from nervous diseases—so many of which are more or less allied with some brain mischief. No, no; whatever cynical economists may urge, and even assuming what they say is true—which it is not—and that they have the right to say it—which they have not, for the people who criticise are generally content with that function—it is a happy thing for our poor sick folk that changing times have brought changes in this respect, and that some outward and visible sign is afforded of the love and sympathy borne them.

Upon passing the vestibule, Mr. Timberlake and his companion found themselves in a spacious hall, from which wide corridors led in different directions. The walls were distempered in tasteful colours; the columns which gave necessary support here and there were of Parian; the pavement was of marble mosaic; and an abundance of stained and tinted glass in the doors and windows gave warmth and colour.

Having inquired for the superintendent, they were soon seated in the office of that gentleman, who readily undertook to make them acquainted with the working of the hospital.

"I," said Dr. Rowe, "am a medical man; and my friend has special and particular reasons for wishing to become personally familiar with the every-day life of the chief metropolitan hospitals, and this among the number."

Knowledge is Wanted.

"I think," said the superintendent, "that all hospitals are glad to show something of their work, and you have doubtless had evidence of that in your round of visits. If we could only arouse a little warmer interest and shed abroad a little more knowledge, the financial embarrassments of the hospitals would soon be remedied."

"I will admit frankly," confessed Mr. Timberlake, "that although I had lived for some years in London and ought to have known of what was going on, I had formed no idea of the value of hospital work until now. I take it, my case is a common one."

"And," remarked Dr. Rowe, smiling, "my friend is still somewhat sceptical about the necessity of a hospital for nervous diseases."

"To my knowledge," answered the superintendent cheerfully, "Mr. Timberlake's questionings have been shared by not a few, whose experience has not brought them into a position to judge. Upon the other hand, those whose opinion is founded upon adequate bases see in the provision made by this hospital a check and an alleviation of the terrors imposed by the last band in the 'grisly family of death.'"

What Need is there for Special Hospitals?

"Under the tuition of my friend Dr. Rowe," said Mr. Timberlake, "I have learned that special hospitals, as a class, are regarded as open to question; that in fact each of them may be fairly asked to justify its existence."

"I quite agree to that," said the superintendent. "We may put it thus: No special hospital is needed unless it relieves a class of patients which otherwise would be neglected. It would be impracticable to deal with fever patients in a general hospital, or with cases of mental disease, or with consumption. In regard to nervous maladies, a particular physician may choose to have cases for clinical purposes, but, as a rule, the sufferers are excluded. The almost stereotyped cry of the applicants and their friends is, 'Our local hospital will not take such cases'; and when we consider the terrible statistics which represent the ravages of these diseases—it is said 120,000 persons perish of them annually in England and Wales—the necessity for the hospital is evident. Then comes the question of ability to do the work undertaken, and to make the best use of the opportunities, because no special hospital puts forward an undeniable claim to support unless (1) it is charged with general knowledge, and (2) it is in a position to acquire and disseminate special knowledge. That is to say, every special hospital ought to be allied as closely as possible with the general hospitals. On these points hear what a very eminent member of the profession has said of us: 'Speciality in this direction is absolutely required. There are admitted into the general hospitals patients suffering from nervous diseases, but in such numbers and for such periods that little or no benefit results to the patient or to science. Here concentration of thought, study, and attention goes on regularly month by month and year by year, and you may depend upon it that better results are worked out and a profounder knowledge of these grave and obscure diseases is here obtained than would be the case in a general hospital.' To complete the case, let me say that we have upon our staff the representatives of no less than nine general hospitals."

"You must have a large staff," said Dr. Rowe.

"Yes; we have five physicians, six physicians for out-patients, and assistant physicians, and six surgeons, besides the resident staff."

"And as to the patients, do they present themselves in sufficient numbers?"

"Here," said the superintendent, pointing to a book he had rung for, "are entered the names of the London patients waiting for admission. Here," reaching from his table a thick bundle of papers, "are the applications of patients outside a radius of thirty miles from London. The morning's post usually brings from six to ten applications by letter, and in the afternoon large numbers of patients attend in person, eager for admission, besides a certain number of urgent cases brought at any hour, and many of the last must be provided for, as the poor people are too ill to be sent away again. When I tell you that our average of daily admissions can scarcely exceed three—that is a thousand in the year—you will see how great is the pressure upon us. Our patients stay three or four times as long as the average patient in a general hospital, and this of course helps to make a block. Then, again, we are called upon to receive a large proportion of country patients."*

No Hospital of the Kind outside the Metropolis.

"Is there no hospital for nervous diseases out of London?"

"Not one. We get applications from the large provincial cities as well as from remote villages, and, I am sorry to add, we don't get any more help from the former than from the latter. Nothing is more common than for those who are generous enough to their own local institutions to overlook the claims of the London hospitals.

"As you propose to take rather more than a superficial glance at the working of the hospital," continued the superintendent, "I should suggest to you to begin at the beginning; that is, with the registration of the new cases of patients. Afterwards we can trace in a fashion the different stages reached by similar cases in the wards."

"That will be interesting," said Mr. Timberlake.

"Then I will ask you to come with me to the out-patients'

* It will perhaps add interest to the narrative if it be noted that the records of figures, facts, and work here given are taken from the registers of the National Hospital for the Paralysed and Epileptic (Albany Memorial).

rooms. Our plan here," continued the superintendent as they walked down the corridor, "is to register all applicants as out-patients, and, as far as space allows, in-treatment is given to those whom the physicians and surgeons certify to be in urgent need of it."

### Subscribers' Letters.

"Do you not think that to insist upon a subscriber's letter interferes with the usefulness of hospitals?" asked Dr. Rowe.

"It would if the rule were too rigorously enforced, but here, as elsewhere, urgent cases are dealt with at any hour of the day or night, subscriber's letter or none. We know that some subscribers are a little jealous of their privileges, but what would be the use of standing out for a 'recommendation' when, for example, a case of apoplexy was brought in?"

"Apoplexy!" cried Mr. Timberlake in genuine amazement. "Is this a hospital for treating apoplectic patients? I didn't know *that* was a nervous disease."

### In the Out-Patients' Room.

They were now arrived in a spacious hall containing comfortable benches for seating from 200 to 250 persons. The greater part of these were already occupied by patients and their attendant friends. Some of the men were reading, many of the women were at needlework, but many more sat motionless, with a weary, painful look upon their faces, pitiable to behold. Patients were continually coming and going with much shuffling of feet and a subdued hum of voices. In front of the benches the space was occupied by an array of wheel chairs—india-rubber tyred and moving noiselessly—each with its occupant, and little rooms opening from the waiting-hall were tenanted by persons on couches. One poor woman lay writhing in all the horrors of a severe epileptic seizure, and moaning drearily. She was attended by a nurse told off for this purpose, and before Mr. Timberlake had had time to look round she had recovered consciousness and soon gave place to another poor sufferer who had dropped to the floor under a sudden attack of the same mysterious malady. At the further end of the hall was a refreshment counter, where a little later on a cup of tea or coffee and bread and butter would be supplied for a penny. This was arranged by a Samaritan Society composed of ladies, who also distribute help in money, food, and clothing to the more destitute patients.

### Quick Work!

"I have seen it stated," said Mr. Timberlake, "that hospital out-patients are often seen and prescribed for at the rate of rather more than one a minute."

"That charge could scarcely be maintained here," said the superintendent. "Half-an-hour is frequently given to a single case, and the patient may be seen by several physicians; anything like rapid prescribing is only usual where the patient has been attending for a long time, and the case is thoroughly known. It is one of the functions of this hospital to keep up treatment for an almost unlimited period in certain cases, and by this means many epileptics who cannot be altogether cured are yet enabled to follow profitable occupations. Competent authorities tell us that the burden upon the ratepayers is much lessened by the work of this hospital, and yet we are called upon to pay £700 a-year for local rates. But, talking of the time expended upon individual patients, of this you may be certain, that there is no case but will be accurately fathomed and diagnosed, so far as science is capable of doing it. We have not here the enormous numbers of patients which attend a general hospital. Our attendances are counted by tens of thousands, where the large general hospitals reckon by hundreds; but in regard to the serious character of the diseases treated, and the length of time treatment is continued, this hospital occupies a unique position."

### The Case of John Harvey.

"John Harvey!" cried the porter, as the gong sounded at this instant, and a wan, weary-looking, emaciated man of about forty years struggled to his feet, with the aid of his wife, who sat by him. He seemed to tremble in every muscle, and his limbs and head gave great involuntary jerks, which sometimes almost overbalanced him, and seemed to threaten dislocation of every joint in his body.

"Now," said the superintendent, "if we pass into the consulting-room with this patient we shall see something of what is going on there."

Mr. Timberlake and Dr. Rowe followed, and found themselves in a spacious room, from which other smaller rooms opened. At the far end, near a large bay window, which let in a broad flood of light, sat the physician of the day, a man of worldwide reputation. In an adjoining room sat the assistant physicians, engaged in taking voluminous notes of all new cases, which were subsequently submitted to the physician. Ranged in a wide semicircle, and listening intently to the remarks of the latter upon the cases brought before him, was a double row of doctors and surgeons, gathered together to witness the practice. On that particular afternoon were practitioners hailing from several of the metropolitan general hospitals, from provincial cities and towns, from India and the Colonies, some Continental cities, the United States, and even such far-off places as Valparaiso and Japan. They numbered about forty in all, every one fully qualified, not a few old in practice, yet, so far as this hospital was concerned, students.

One or two who had attended regularly for some time were engaged as volunteer assistants in using the ophthalmoscopes, or testing muscles by means of electrical batteries; others were in the adjacent examination-rooms still further elaborating the notes made concerning cases passed over to them for the purpose by the physicians.

Every new patient after such notes had been fully completed came before the physician, who scrutinised both the notes and the patient, said something about the case to the listeners about him, and, having added his own observations to those already recorded upon the patient's papers, either prescribed for him as an out-patient, or when necessary wrote a recommendation for his admission to the wards.

The double row of onlookers all craned forward, as poor John Harvey was placed in a chair, where he had to be held by his wife and a friend to save him from falling to the ground.

His was one of the painful stories to which humane hearts never grow accustomed, be they repeated never so often, and the physician shows plainly he is not yet steeled to indifference.

A story of an educated man's worry and overwork—John Harvey was a schoolmaster by profession—of terrible misfortunes and anxieties, with desperate efforts to retrieve his position, and then the final breakdown.

"Would you like to come in?" asks the physician. "You are hardly fit to go back home to-day."

The husband and wife exchange glances—his so hopeless in appeal—hers so sad, yet full of resignation.

"As you think best, doctor," he gasps with an effort, and the vibrations of his poor limbs are dreadful to witness.

The needful words are written, the gong sounds, and John Harvey is helped away.

### John Harvey in the Ward.

Half-an-hour later, when Mr. Timberlake and his companions reached the ward whither he had been conveyed, they found the poor schoolmaster already in bed, and—now he was recumbent—free in a measure from the terrible spasmodic movements which never cease when he is upright.

His wife, leaning over the bedside, holds one of his wasted hands in hers. "You will try to be hopeful, dear, won't you?" she whispers. "God is with us. He will yet be merciful."

The husband nods his head without moving the glance of his sunken eyes from her face. She stoops to kiss him, and then moves away—his look hungrily following her. She turns in the doorway, kisses her hand, and is gone.

"What is it?" asked Mr. Timberlake.

"Chorea," says Dr. Rowe; "the somewhat familiar 'St. Vitus's dance' we associate mostly with children."

"Yes," said the superintendent, "in the case of the children the disease seems tractable. We get many who suffer from it, and they generally go out cured; but in adults the causes are doubtless different and more grave, for the doctors get many disappointments."

The dormitory in which they were standing was a bright, cheerful apartment. The walls were distempered in French grey, and a high-pointed dado of a darker colour preserved them from soiling. It was a peculiarity of this hospital that each "ward" consisted of two chief apartments, a day-room and a dormitory, besides a ward kitchen, bath-room, and ward offices. The ward contained eighteen beds, and was fully occupied. Just twelve beds had tenants; the other six patients were able to sit up in the day-room. There was a multitude of good engravings, and some even more ambitious pictures were hanging on the walls. Above the heads of several of the beds were marble tablets let into the wall. These bore inscriptions, and denoted that the particular beds were either endowed or maintained "In Memoriam."

John Harvey occupied one of the latter, at which the superintendent expressed satisfaction.

### The Perfection of Philanthropy.

"For," said he, "the lady who maintains that bed feels special sympathy for those who have been brought low by their illness, and so I always try to keep it tenanted by patients who have seen better days. Not one of them is forgotten by their benefactress, and although she is an invalid herself and cannot come to see them, she writes regularly and sends illustrated papers and gifts of beautiful flowers week by week throughout the year, to show how she holds them in remembrance. And she keeps up the acquaintance after they have left us, so that every poor fellow who has occupied that bed knows well where to turn for a friend."

"That seems the perfection of philanthropy," said Dr. Rowe.

"Yes," said the superintendent; "she not only helps the hospital, but in addition she identifies her sympathies with individual suffering, so that they are always finding fresh nutriment."

In the centre of the ward was a long polished table, profusely decked with flowers, to which the superintendent drew his visitors' attention, as some of the choicest were supplied by the lady just spoken of.

"It is astonishing what delight the flowers give both to patients and nurses. I scarcely know what the hospital would be without them. A little blind girl once said, 'How I wish I could see the flowers, nurse! But,' she added cheerfully, 'I can smell them, can't I? And the scent tells me what flowers they are.'"

### A Terrible Case.

"What is the matter with that poor boy?" asked Mr. Timberlake, pointing to a bed from which the bolster and pillows had been removed, and which contained a lad of fifteen lying motionless upon his back, with a bandage passed round his chin and attached to the head-rail of the bedstead.

"A fracture of the spinal column high up towards the neck, and he must not move his head. He has been lying in that way for a month."

"Poor boy!" said Mr. Timberlake, "is it possible?"

"Yet he greets everyone with a smile," said the superintendent, "and is inclined to be loquacious. The case in the next bed is similar. The man fell from a window he was cleaning, and bent his back over a ridge on the leads below. Both cases have been surgically treated; the splinters of bone which pressed upon the spinal cord have been removed, and time will show whether power will be restored."

"You seem to get a fair share of surgical work," said Dr. Rowe; "more than I should have supposed."

"Yes; of late especially. For some time the hospital was almost wholly medical in its character, and what surgery we practised was chiefly orthopædic. Now we get considerable variety, including some of the gravest operations within the range of science."

"Yes," said Dr. Rowe, "those marvellous brain operations I have read about. By the way, now I am in town, I should like to witness some brain surgery if I could."

"There will be an operation for abscess to-morrow. The patient was only admitted to-day, and it is therefore unusually quick work; but the man is comatose. Generally, the doctors like to have the patient under observation for some little time."

"Could we see the patient as he now is?" asked Mr. Timberlake.

"Certainly, if you will come upstairs to the ward above."

### The Man with the Abscess on his Brain.

Having reached the ward in question, the superintendent stopped at the foot of a bed whereon lay a man apparently moribund; there was the movement of heavy, irregular breathing, and a slight quivering of the lips, otherwise he was as motionless as unconscious.

"He has lain so for a week, the wife tells me," said the sister coming forward.

"Is there any external mark of injury?" asked Dr. Rowe.

"None," replied the sister, glancing at the patient, whose head had been clean shaved within the last hour.

"In some cases," said the superintendent, "there is a history of accident; but in this I believe there is nothing of the kind."

"I don't quite understand," said Mr. Timberlake, who was looking with undisguised feeling upon the poor helpless creature before them. "What is it that is to be done?"

"Well," said Dr. Rowe, "they have determined by certain observations, rapidly made in this case, that an abscess is pressing upon the brain, and that the abscess is the cause of all the mischief. To-morrow the cranial cavity is to be opened and the abscess removed."

"Surely it is a terribly dangerous operation," said Mr. Timberlake.

"True, it is a grave proceeding; but I understand your surgeons have been wonderfully successful."

"Yes," replied the superintendent; "and in many instances the operation has not only been successful in itself, but in its results."

"Is it now thought that all brain tumours can be treated surgically?" inquired Mr. Timberlake.

"No; but we do not know of what science may be capable in the future. Happily, some tumours may be dispersed by medical treatment alone. Thus, a little girl left us last week quite well and bright. When she came in, two or three months ago, she was blind. A brain tumour was diagnosed, which was quickly got rid of by the action of drugs."

"And did she recover her sight?"

"Perfectly. When we reach the ward for children we can see another very similar case, with an equally satisfactory termination. An operation of so serious a character as that we are speaking of is only resorted to when it is morally certain that without it, life—or all that is valuable in life—is already doomed. This poor fellow, as I understand from the medical officers, if left alone, will never recover consciousness, and must die after lying at most a few weeks. Well, the position has been clearly explained to his wife and brothers, and they know what the chances are.

"Can we see any patients who have passed through similar operations?"

"Yes, we have three with us now. Here is one. Now, Pargeter, tell these gentlemen how we've treated you here."

### Pargeter's Statement.

The man addressed looked up cheerfully.

"Oh, sir, I don't know what they did. I know nothing about that; but I know that all the dreadful pains in my head have gone, and Dr. —— says I may get up next Sunday before my wife comes."

"Pargeter is in an early stage yet," said the superintendent; "only about three weeks have gone by since the operation. But here is another patient who has got over three months."

The man referred to was sitting at the table, but rose to his feet as Dr. Rowe went up to him, though the latter soon made him take his chair again. He was wearing a sort of flat leather helmet to protect his head from injury, and though his face bore unmistakable traces of past suffering, he seemed perfectly well, though weak.

"Ah, gentlemen, I never thought to get out of bed again, I can assure you; but as the good gentleman the chaplain says, God be praised for all these kind doctors and nurses have done for me. I can't bless 'em enough, sir."

"I notice," said Mr. Timberlake to the superintendent, looking round the ward, which in size and arrangement was a counterpart of those already visited, "there seems a great variety of diseases. I could never have supposed there were so many types of nervous maladies."

"A distinguished physician," answered the superintendent, "tells us that there is paralysis ranging from weakness in a finger up to death in life of the whole body, and epilepsies which range from 'weird seizures' to 'convulsive explosions,' sudden and terrible, as though the brain were made of dynamite. Now here is a patient representing the 'death in life.' He has been with us many months, and lies there still—blind, deaf, dumb, and without the power to move a single limb."

"Can anything be done for him?"

### Death in Life.

"I take it, the case is one absolutely hopeless, but the poor fellow is just kept alive by attention. You may perhaps ask, *Cui bono?* I am tempted myself sometimes to ask that question. Still, it is the duty of the hospital to neglect nothing which can keep life in the body, and if that man were sent to his own poor home, or to any place where he failed to get all he gets here, he would die at once; nay, I am told by the doctors, because reports are always required concerning these 'overtime' cases, as we call them, that even the journey would in all probability kill him before it was over."

"And possibly," said Dr. Rowe, "though you cannot put him on his legs again, he may teach something useful to know, which may help to benefit others."

"Just so; it appears to me quite a legitimate function of the hospital to tend a sufferer in his last days, even though they are prolonged. Our difficulty is, that such cases occupy beds to the exclusion of those more hopeful. Talking of varieties of illness, let me draw your attention to the papers of the patients in this particular ward. There are here, of left-sided paralysis, two cases; of right-sided paralysis, two cases; of double paralysis, three cases; tumour on brain, three; shaking palsy, one; hardening of the spinal cord, two; muscular atrophy, one; cerebral meninigitis, or inflammation, two; sciatica, one; neuralgia of facial nerves, one. If we were to go right through the hospital, we should find maladies almost innumerable."

### Aphasia.

"I suppose," said Dr. Rowe, "some of the patients are aphasic?"

"Yes; more than one in this ward."

"What is that?" asked Mr. Timberlake.

"Aphasia," replied Dr. Rowe, "is a curious condition brought about by some lesion of the brain, generally softening, or it may be due to some functional disturbance. You see the brain is now, so to speak, mapped out, and every part is accorded its own particular functions. Thus you cannot move a finger without the excitement of the particular group of nerve fibres which command it, and consequently if that group be injured or destroyed you lose more or less completely the use of the finger. When the injury extends to several groups paralysis affects the corresponding parts of the body, and if the nerve current is wholly intercepted by, for example, some injury to its channel, the spinal cord, you have absolute paralysis below the point of injury. Among the rest there are parts of the brain termed 'speech centres,' and when these are affected, all kinds of strange results follow attempts to speak. Words may be used to express a meaning precisely opposite to the intention, or they may be ranged in order absolutely meaningless, or, in some cases words cannot be articulated at all. It is just as if one possessed the thoughts of a man, but had no more power than a baby to command language."

"We have had patients here," said the superintendent, "who have lost all ability to read, although they have remained able to write; and one curious case is recorded where the patient could copy in facsimile, but could not write a single word spontaneously or from dictation with his right hand. Nevertheless he could write easily with his left, and then with his right hand copy what he had written."

"Here is a man suffering from insomnia. He thinks he never sleeps, but that is of course a mistake, though it is only at long and uncertain intervals."

The poor fellow raised his head and looked piteously from one to another of the group.

"I can't get a wink of sleep, gentlemen; indeed I can't. I have been awake ever so many weeks. Oh, my head! If only you would give me something to make me sleep, sleep, sleep—for I am so weary."

And he sunk back on the pillow as though exhausted.

### Massage and Electricity.

"You have a great deal of shampooing and rubbing here, have you not?" asked Dr. Rowe.

"Yes, probably more than anywhere else. It is quite a business; all our own nurses are taught, and we also receive nurses from other hospitals who go through a course of training here in massage and electrical work. They give their general services for three or six months, and we supply them with special instruction by means of practical demonstrations in the wards, and lectures delivered by members of the visiting and resident staff."

### The Cure for Neuralgia.

"Did you just now speak of facial neuralgia?" asked Mr. Timberlake.

"Yes; we get many of the most obstinate forms of that. The patients seem to come to us after everything else has been tried. We have a farmer's wife here now who suffered intense agonies almost continuously for twenty-eight years. She will go out this week cured."

"What was done?"

"The nerves were excised, and there has been no pain since. The poor woman is overcome with thankfulness, and her friends regard it as a miracle."

### Discipline and Work.

Upon moving from this ward to one beyond, the change was a little startling. Entering the day-room, a number of active youths and boys were discovered seated round a large table, taking tea, with huge slices of bread and butter and bread and jam. At the head of the table sat an attendant in neat uniform, and smart as an old soldier should be. As the visitors came in the attendant brought his charges to their feet in an instant.

"The maintenance of discipline in an epileptic community is half the battle," said the superintendent to the visitors, "and we always therefore demand much in that way of these particular wards. Sometimes a patient is admitted with a history of frequent fits, which cease directly he becomes subject to the control and regularity of hospital life, and before he has swallowed medicine of any kind."

The boys were now motioned to proceed with their meal, which they did with alacrity. Evidently appetite was not lacking. Indeed, as the superintendent said, the difficulty is to satisfy some of them.

"It does not appear," said Mr. Timberlake, "as if they had much the matter with them."

"Sometimes," said the superintendent, "it seems to me they are more to be pitied than those who appear more heavily afflicted. They are not unlike frail vessels sailing upon seas where squalls abound."

### A Case of Convulsions.

As he spoke there was a considerable clatter and hasty thrusting back of chairs from the table, and as the other patients move away the attendant is seen supporting the head of a strongly-built youth, who is in violent convulsions. The cup, which it appears he was just lifting to his mouth, came rolling along the ground, his plate and saucer lie under the table.

"Fortunate it was not earthenware," said the superintendent, picking up the cup. "We used to have an enormous number of breakages in the wards at one time, but now we use what is called 'granite ware'; it is scarcely possible to break it."

Meanwhile one of the patients had brought a pillow, and, after a writhing movement of the body, continued for a few moments, accompanied by a grinding of the teeth, loud moanings and violent twitchings of the face, the boy suddenly looked round him with a dazed expression, and became perfectly still. He was then carried to his bed, and laid upon it.

"That is a terrible sight," said Mr. Timberlake. "How long time will elapse before he recovers full consciousness?"

"That boy," answered the superintendent, after questioning the attendant, "soon revives, but sometimes the effects last through two or three days. In the same way, the seizure itself may end in a few moments; it may be a 'sensation,' as it is termed, or the patient may be struggling, foaming, and unconsious for half-an-hour. Scarcely any two cases are precisely alike. Some have their fits at regular intervals—daily, weekly, or monthly; some in batches of a dozen in a day, and then none for a time; some have them by night always, and some by day; some know perfectly well when an attack is impending, others are taken without warning of any sort."

"Of course," said Dr. Rowe, "all the circumstances of each case are carefully noted."

"Certainly. We have electric communication from every ward to the rooms of the house physicians, and instructions are given in particular cases to nurses and attendants to summon the doctor directly a fit begins. I have known the doctors waiting for weeks to catch a patient in a fit in order to note certain phenomena."

"But, of course," said Dr. Rowe, "neither the house physician nor the members of the staff can see everything for themselves, and I take it your nurses are trained to use their eyes."

"Just so, and now more than ever. Every incident, however trivial, is carefully recorded in writing. By day and by night the same unrelaxed observation is maintained, and every look and act and movement of the patient during the progress of the fit is set out."

"Truly epilepsy and its kindred ailments are among the most mysterious of maladies. But what of the patient we were to see; is he in this ward?"

### The Patient with the Helmet.

"Yes; we may know him by his helmet."

The patient wearing the strange head-gear came forward at a sign, and answered the questions put to him with great vivacity. He was hoping to go out soon, he said, for his physician had told him he thought he might.

"Ah," said the superintendent, "we gave him and two companions a trip to Brighton the other day, to make the acquaintance of the British Medical Association, and they were honoured by an article in the *Times*, telling the whole world about them."

"What was the patient's condition when he came in?"

"He was having continual fits. Two attendants were told off to look after him, and he was watched night and day by one or the other of them. A thousand fits had been recorded in less than a fortnight, and the man was at the point of death, and, humanly speaking, must have died within a few days."

"Good heavens!" cried Mr. Timberlake.

"That," said the superintendent, "was the exclamation of another visitor, who added that if he were a physician he should feel inclined to put the poor fellow out of his misery. Well, it was determined to open the skull."

"How," asked Mr. Timberlake, "do they know where to begin?"

"That is mathematically determined by previous observations. Abnormal indications supplied by certain nerves are traced back to the particular nerve-centre involved. Science enables this to be done now with the utmost accuracy."

"It would never do," said Dr. Rowe with a twinkle, "to be making holes in people's skulls on the bare chance of finding what they were in search of."

"When the operation in Blake's case was performed," continued the superintendent, "the surgeon discovered, at the precise spot indicated beforehand by the physician, a piece of diseased brain tissue, which he removed."

"And the result?"

"Within twenty-four hours the patient was able to converse rationally—was a little too eager to talk, in fact—and in ten days he was back from the surgical ward, and truly 'put out of his misery,' but in a different sense from that intended by the too sensitive visitor."

"Have there been any fits since?"

"Not one. Of course this is so far one of the most successful of these cases. Some patients are apparently cured, and in other cases the result has been great mitigation of suffering. One poor man, after being operated on, recovered his full faculties for three months, then the growth recurred, and he was re-admitted—to die."

### The Cost of an Operation.

"Even in that instance," said Dr. Rowe, "there was the distinct advantage of a respite. These must be very costly operations."

"Exceedingly so; antiseptic surgery is always expensive. Every operation is calculated to cost the hospital £10 in special requirements over and above the ordinary outlay on a patient."

"And I suppose yours is a costly class of patients at all times."

"In some items, yes; in others, we are perhaps below the general hospitals. We have a very large proportion of nurses—very nearly one to every three occupied beds; then some are male nurses, and they are expensive; and besides that, we want more than the usual number of porters, as we deal with so many helpless people, and our lifts are always moving. Again, we have a larger cubical space allotted to each bed than is the case in any other hospital, and that means more scrubbers and ward-women. On the other hand, our diets are, as a rule, less varied; and 'extras,' though heavy enough, do not reach the dimensions they do elsewhere."

"Does the subscription representing a letter bear any relation to the cost of the patient?"

"Not any, practically — less here than anywhere else probably, because of the longer average time a patient is kept. The cost of each in-patient admitted last year was about £14, and in many cases we do not get a subscription. Patients are sent to us in a condition which compels their admission, for they are often too ill to go back again. The clerks come to me frequently bringing papers with some such story as this: 'Urgent—ought to be admitted at once.' 'Tell Dr.—— with my compliments, that we have not a bed.' The message comes back, 'The patient is not fit to be sent away, and Dr. —— says he must leave the responsibility to you.' What is to be done then but admit, even though a bed must be taken from some other patient, who is forthwith consigned to the sofa or the floor."

### PAYING PATIENTS.

Passing onwards, the trio next entered a ward for patients who contribute something towards the expense of their maintenance. "We are careful," said the superintendent, "as to the patients we admit to this ward, for there are many well-to-do people who would come without compunction to take what is intended for the poor. Each patient contributes about two-thirds of the actual cost of his food, nursing, medicine, etc., while they get the medical attention and all the advantages of the hospital free."

"And by way of illustration, what is the social position of some of the patients here now?"

"One is a country clergyman, one a doctor broken down by over-work, two have been schoolmasters, several are clerks, one is a small farmer, and so on."

"Do they get anything more than the free patients?"

"Well, whether free or contributing, they would get all that is necessary—no matter what the cost. A little more privacy and not quite such mixed company comprehend the chief points of difference; but I assume the patients feel a real satisfaction in doing what they can to meet some part of the expense, so that they are not wholly a burden upon the charity."

They were now in another ward of eighteen beds—the latter mostly occupied, but some few patients were in the day-room.

A very helpless community it seemed: some were being fed by the nurses, several lay apparently oblivious of everything; one or two were propelling themselves along the ward in wheel chairs, stopping here and there to have a word with a fellow-sufferer.

### FROM FAR AND NEAR.

"I was telling you just now," said the superintendent, "that the country shares directly in the benefit of London hospitals. I think this ward offers an almost unique illustration of the truth of that. Will you notice where the patients come from?"

The address was set out upon the bed ticket which hung at the foot of the bed, and as they passed down the ward the superintendent read out the county represented. The first was Essex, and then in the order named: Monmouth, Lincoln, Warwick, Surrey, Sussex, Lincoln, Middlesex, Westmoreland, Lancashire, Somerset, Lancashire, Yorkshire, Carnarvon, Devon, Kent, and Dumfries. Patients from fifteen counties in all, and only one Londoner among them.

"Yet," said the superintendent, "when appeals are addressed to country residents, they often reply reproachfully that their local institutions need all they can give."

### RUSSELL AT LAST.

When the county of Devon was named, Mr. Timberlake bethought him of his neighbour, and asked if the patient's name were Russell, and did he come from Northhoe, and was he paralysed in both legs?

"The same," said the superintendent; "he came in about a fortnight ago."

As the bed allotted to Russell was empty, Mr. Timberlake hurried back to the day-room. Russell was not there, however, and the sister explained he had gone to the electrical room.

"Oh, well," said the superintendent, "we can take that on our way; I think you may find it interesting."

### THE ELECTRICAL ROOM.

The electrical room was adjacent to the department for out-patients, and was of fair size, though the floor space was considerably diminished by the formidable array of apparatus. The chief agent made the least show, perhaps, as it appeared to consist of little more than a few insulated wires connected with a table, but these wires came direct from a chamber below, bringing to the service of the physicians the powerful current of electricity produced by the working of a steam engine and a dynamo. On the table was fixed the resistance coil, through which the current of electricity, fresh from the dynamo, passes, and is, as it were, filtered in its passage. After that the current makes its way through a dynanometer, which both measures and indicates its power, and then, regulated by a simple contrivance, it is poured through the rheophores into the patient's limbs or body in quantities as accurately adjusted to each patient's strength and need as though they were doses of medicine.

Besides this, there were portable batteries of different sorts and capacities, static machines which gave off easily a five-inch spark, insulated sofas and chairs, with baths of different sizes and shapes for feet, hands, and arms.

Passing behind a screen in wake of the superintendent, Mr. Timberlake and Dr. Rowe found their sought-for and much-startled neighbour. He was scarcely in a condition to receive visitors, for he sat unclothed to the waist, the attendant being engaged in holding one pole to the back of the patient's neck, while with the other he gently stroked the patient's back. The superintendent apologised to the doctor for the interruption, for hospital etiquette demands that as far as possible doctors and surgeons shall not be intruded upon while with their patients. Dr. Rowe and Mr. Timberlake, having warmly greeted their neighbour, and having been informed by the doctor that he was making a good recovery, being already able "to feel his legs" and to walk across a room with the help of a stick, said they should take care to convey the good tidings to his wife, and left as delighted at having unearthed poor Russell as he was to receive a visit from them.

"Have you many patients under electrical treatment?" asked Dr. Rowe.

"I suppose about one-sixth of the whole number of in-patients receive electrical treatment of one sort or another. The attendances of out-patients in this room numbered about 4,000 last year. Most of the in-patients are treated in the wards. Of course it is not only in treatment that electricity is valuable; it is very largely used in diagnosis, and by its means much is revealed that otherwise might remain hidden."

"Exactly," said Dr. Rowe; "science has learned to rely less on symptoms than was once the case. I observed how many lamps for ophthalmoscopic work you have in the consulting-rooms."

"Yes," answered the superintendent; "we are told the eye is the index to the brain, and that the physician is now able to detect with absolute accuracy the existence of lesions which formerly could only be guessed at. This, of course, accounts for the many cases sent in here from the ophthalmic hospitals; patients go to them for what they consider an eye affection, and upon examination it may turn out to be due to some brain mischief, altogether unsuspected."

### The Hospital Chapel.

Coming along the chief corridor, the visitors noticed the subdued and not unpleasing sounds of the chapel organ. Looking in, they found one of the sisters playing the instrument, and a group of nurses off duty stood round practising a simple anthem.

The chapel was small but beautifully designed, and looked rich in colour as the sun's rays shone through the handsome dome of purple and gold, and having absorbed some of the tints, cast them, diluted, upon the painted walls and the snow-white head-gear and aprons of the nurses.

"We are all very fond of our little chapel," said the superintendent, "and I think the mind of our good chaplain is never quite free of his work here. He gives us a great part of his time, and is always ready to help in every way. Next Sunday will be Hospital Sunday, and we shall muster in full force. Every patient who is fit to leave the wards, and willing, will be here, and we look for quite a full congregation."

"I should like to come," said Mr. Timberlake, "if I may."

"Certainly; and though our service will be plain and short—as it must be, considering all things—I will promise you this, that you shall scarcely find a more devout or sympathetic congregation."

Leaving the chapel, the superintendent conducted his visitors through the chief hall, pointing out to them the tablets which recorded the early history of the hospital and its gradual growth and development. There were stained windows too, which displayed various legends connected with the progress of the institution. Then they descended to the basement, and traversing a long corridor—from which opened out on the one side a range of store-rooms for hardware, crockery, groceries, bread, wine, beer, &c., and on the other refectories for nurses and refectories for servants, housemaids' pantries, and other apartments—they passed the general store where the different articles for consumption in the wards and other parts of the hospital are issued. Everything is weighed out and apportioned in accordance with a fixed scale, and the several items are checked off and balanced almost as accurately as though they were money instead of tea and coffee, sugar, soap, wines, spirits, &c.

### The Question of Stimulants.

"Do you use much wine?" asked Dr. Rowe.

"I think rather less than the average elsewhere. The tendency everywhere is to lessen the consumption of wines and spirits. In some of our wards scarcely an ounce of either is consumed for days together, and it goes without saying that none is given out except upon the prescription of the physicians. To officers and servants who wish for it, beer is supplied at the rate of a pint and a-half a-day, but many prefer milk. As to economy, it makes little or no difference which is had; they cost about the same."

### In the Kitchen.

Entering the kitchen, the visitors noticed the quiet and cleanliness. The doors of the ovens shone like ebony, and the gun metal cornices and bands were as brilliant as leather and rubbing could make them. So too were the copper lids of the formidable row of boilers, and the balancing weights and chains by which they were held open. So too were the mighty ranges of dish-covers, colanders, and other metal utensils, which hung so thickly in the scullery, it looked like an armoury. As to the line of sinks, they were clean enough to eat out of, as people say; and the wood-work, the hot plates, and the tiled floors were spotless.

"In the matters of cleanliness and lessening of labour," said the superintendent, "there is a great advantage in cooking by steam and gas. The steam and hot water for the kitchen, wards, and building throughout—including the heating radiators—are supplied from two Cornish boilers, and the engineer in charge can turn on or withdraw warmth, water, or steam from any particular part or the whole by turning a handle."

"Is there any need," asked Mr. Timberlake, "for heating corridors and passages."

"Yes; in winter it prevents the sudden rushes of cold air when doors are opened, and continual draughts, which are otherwise unavoidable in big buildings. It is of great consequence that as far as possible the hospital throughout should be kept at an equable temperature, for not only are patients frequently conveyed from one point to another for baths, electrical treatment, and other needs, but as the nurses for obvious reasons are not allowed to make any outer addition to their uniform clothing, they would suffer greatly, especially at night-time."

From the kitchens the visitors went to the boiler-room, then to the large bath-room and accessories, containing every variety of bath needed for medical purposes, including douche, shower, plunge, sitz, needle, Turkish, and sulphur baths—the latter much used in cases of lead poisoning, dropped hands, etc.

Then mounting to the ground floor level, they passed the entrance to a terrace and open-air court, with a few trees in it. Several young patients, convalescents from the wards, were at play, and some others, older, were seated round about. The visitors stood leaning over the balustrade for a moment to watch them.

"These open spaces," said the superintendent, "are of immense value, and I wish we had more of them; but gardens and courts are difficult to provide in London, and when you have them there is trouble in coaxing the plants to grow. At our country home, the garden, a well-timbered, old-fashioned place, is the chief source of delight. Here we have to be satisfied with few shrubs and plenty of asphalte and terra cotta.

### The Country Home.

"How many beds have you at the country home?"

"Twenty; and in summer we want as many more, because many of our patients are in-admissible to the ordinary convalescent institutions. Few people can realise the pleasure a glimpse of green leaves gives not only Londoners, but also our country patients. It seems as though the trees give pledge that they are still in the same world which holds their own quiet homes far away.

### Surrounded by Trees.

"Here, though we are in the very middle of the metropolis, we are almost surrounded by trees, and they are greatly loved by the patients. One range of wards commands the view of a grand old sycamore standing in an adjacent garden, and many a poor fellow whose bed is on the wrong side of the ward hears what his opposite neighbours say, and regrets he cannot get a view of it."

"'Why, sir,' said one from a little village in Northamptonshire, 'we've got a tree just like that in front of our cottage. I lie and watch it, and think of the old place. You see that branch bending over like. Now it's got all its leaves, I can't see a chimney-pot anywhere; its just like the country, with the blue sky, and the leaves blowing about, and nothing else; isn't it, sir? They do tell such tales to us country folk. I thought there was no sun or trees in London—nothing but smoke and noise; and my neighbour Tom Harness says—why, he says the smells are enough to kill 'un; but I don't see anything to complain of; and as to noise, why there ain't a quieter place than this as I know of, let alone the birds, which I think are more twittering like than they be with us.'"

Flower Shows in the Hospital.

"On grand days here," continued the superintendent, "we put up tents on this terrace and beyond, and have rose and flower shows. They are very attractive, and we get together a great many visitors, and some of them become interested in the place, and remain good friends afterwards. We always arrange that the patients shall have the whole place to themselves for an hour or so, and last summer many of them, who had never seen a flower show in their lives, seemed to think the fairies must have been at work, or that they were dreaming."

Continuing their way, the visitors came to the wards for women, and passed into one which bore the name of a lady who, as the superintendent explained, had been a great benefactress of the hospital.

A Typical Case.

"Now, here," said the superintendent, "is a patient whose case furnishes a good illustration of the hospital work, sketched in bold outline. There has been protracted and varied treatment, both medical and surgical; and the result, as gradually brought about, is all that can be desired."

The patient referred to was a young woman, looking now the picture of health and intelligence. She had been in the ward no less than a year and eight months. When admitted, suffering from what the doctors call diffused myelitis, she was prostrate in mind and body—paralysed in all four limbs, her legs terribly contracted, and apparently she had but a short time to live. Bedsores which were forming when the patient was admitted rapidly developed, notwithstanding she was placed at once upon a water bed, and all the usual precautions were taken. The first step gained was to bring back the flesh and skin to normal condition."

"Do your staff," asked Dr. Rowe, "consider bedsores preventable by good nursing?"

"I dare say, in most cases—but not in all by any means. The condition of the flesh and blood is such in some instances that it is impossible by any care to prevent sores forming. I have been told of cases where the resting of the finger tips on the sheet produced a sore on each finger within a short time."

"Ah, in your hospital education, Timberlake, you must not overlook bedsores—the frequent accompaniments of paralysis. Anything more dreadful than the condition of some patients, especially when aggravated by neglect, can scarcely be conceived. Often the sore will eat away the flesh to the bone beneath."

"Good gracious!" cried Mr. Timberlake.

"Yes," said the superintendent, "we get many cases admitted in the state you describe, and the task before the doctors and nurses is almost appalling. But to return to our patient here. Several months were spent in recovering the use of the arms and hands; meanwhile, the contracted legs were taken in hand, extension weights were used, plaster of Paris bandages followed, then the surgeon stepped in and divided different tendons by three distinct operations at intervals, then special instruments were made and fixed, the legs were straightened at last, and now, final triumph for physician, surgeon, and nurses, the patient not only has full use of her upper limbs, but she is able to walk about the ward, and (turning to the patient) we hope she will now very soon walk out of the hospital, for I think the doctors have almost had enough of her."

The patient smiled at this, and promised to do her best.

"The moral of this case," said the superintendent, "is to be found in the long, tedious, and expensive processes of treatment employed. Athough a distinguished physician has said that modern miracles are performed here, we do not lay claim to anything more than the power to apply all the resources of science in behalf of these tedious cases, and to give the patients the full time needful for the successful treatment of their obstinate maladies. The cost to the hospital of that particular patient cannot be less than £150.

"Of course," said Dr. Rowe, "that must be an extreme case, both in regard to the time spent, and the varied nature of the treatment."

"Yes, but it is not an infrequent occurrence for a patient to have a hospital birthday with us—that is, to complete a year in the ward—and we must have at least a dozen with us at this moment who have undergone from six to eight months' treatment."

Passing down the ward, Mr. Timberlake remarked upon the number of painracked, careworn faces.

"The separation from their home," said the superintendent, "seems to be an abiding trouble with many of the women; it is not, of course, confined to them, but they seem more generally depressed than the men."

The Medicine of Work.

This observation scarcely applied to the ward they now entered, for every patient in the day-room was looking quite bright and cheerful. With only three exceptions they were busily and usefully occupied.

"A physician formerly on the hospital staff," said the superintendent, "has recorded his opinion that idleness is the curse of epileptics, and every day we prove the truth of that assertion. This is the most industrious ward we have. All the linen is mended here, new sheets are hemmed, blankets are overstitched, marking is done, and last, perhaps not least, when a pressure of office work occurs, they often give the clerks downstairs many helping hands in folding, stamping, and sending letters and papers for the post; indeed, I do not know what we should do without them."

"And some get quite cured, do they?" asked Mr. Timberlake.

"Yes, many; but the future alone can show what amount of benefit has been gained. I often receive letters from all kinds of out-of-the-way places, abroad and at home, referring to the writers' stay in the hospital, and stating they have been well since. Sometimes they have married, and sometimes they are holding good situations, and often they want to help some one who is afflicted as they once were. One poor man the other day sent ten shillings as 'a gift from a patient of 25 years ago.' Another patient has informed me she has bequeathed all she possesses to the institution; another writes that she never neglects to pray for the prosperity of the hospital which cured her. It does one's heart good to read these letters, and I can say of my own knowledge, yes, these sufferers are often perfectly cured."

"And what becomes of those you can't cure?"

"Heaven and their friends only know. In all the records of suffering, I can imagine few more painful passages than that which might be written of the woes of incurable epileptics, yet sometimes I think that the silence which enwraps their fate is even more expressive than words. Talking of incurable sufferers, we are peculiar in possessing a

Pension Fund for the Incurable."

"And," said Dr. Rowe, "I fear you have also peculiar need of it."

"Alas! yes. No hospital deals with a more intractable class of maladies; and science, although always advancing, is not yet equal to all the demands made upon it."

"Is the Pension Fund a charge upon the hospital?"

"No; we have not the power to divert a single penny to its use. Each pension has been fully endowed by some good donor. How greatly the pensions are valued may be judged by an incident I have heard of to-day. A poor man who had been waiting in expectation some time has actually died of excitement and joy because he received the form of application."

"Gracious goodness!" cried Mr. Timberlake.

"Yes, here is his widow's letter. 'In the deepest sorrow, she writes 'I beg to return the papers sent to be filled up by my dear husband, who, I must tell you, passed away on Tuesday. The poor thing was quite overjoyed when the paper reached him . . . but without a moment's warning the dear fell on the floor quite dead.'"

"Think of that, Rowe," said Mr. Timberlake, with deep feeling, "think of that—to die of joy at the bare chance of getting £10 or £20 a-year! Who would suppose such things possible?"

### The Children's Ward.

Hereabouts they met the matron of the hospital, who, after introductions, inquired with some little sarcasm, whether the superintendent had forgotten his engagement, and this led to an explanation that he had undertaken to drink tea with the inmates of the ward for children; who, it appeared, were then occupied in a festive celebration of the silver wedding of their Royal Highnesses the Prince and Princess of Wales.

Apologising for his neglect, the superintendent suggested that the matron should invite Dr. Rowe and Mr. Timberlake to share the hospitality of the little ones.

The ward was a scene of much excitement, giving a chance comer but a poor illustration indeed of the gloom and gravity some people associate with the inside of a hospital. The laughter of the little inmates, the positive shrieks of delight with which they were greeting the final episodes in that time-honoured drama, "Punch and Judy," reached the party of visitors as they hurried up the staircase, and seemed to give the lie to the records of the dire maladies many of them suffered.

Prominent in the group was the chaplain, the lithe and active sisters and nurses, and a few convalescent patients from other wards, with a sprinkling of visitors, making up a goodly assembly.

Of the twenty-five cots, everyone festooned with flowers, fifteen had turned their occupants loose for that evening at least, and of the remainder, all but three contained laughing, happy little faces, and forms tremulous in an ecstasy of gladness. All but three! And what of those they held? Poor little stricken ones; they lie there oblivious of everything—neither soothed nor disturbed by the unwonted bustle about them—only unconscious of it all.

Two of them blind and deaf, one of them blind, deaf, and dumb, all three paralysed. And the cause? Some tumour or abscess pressing upon the brain, which seems to annihilate all a healthy child would understand by life and yet to rob Death of his prey.

And what subtle influence, what mysterious bond of sympathy is that which causes a smile to pass over the little face when nurse comes up—his nurse, her nurse; surely that much is present to the buried mind—and takes the little hand for a moment?

So surging round these three poor isolated ones, the frolic goes on, and Jacky, who is the contortionist of the ward, and can only get about by shuffling on his knees to the woeful destruction of his stockings—and is going to be operated upon and put up in splints shortly—moves in and out among the legs of the company as happily as though he were as straight as Adonis and had every reason to take the very brightest view of life.

Then the sisters distributed oranges and lemonade—champagne, the knowing ones call it—and someone proposes "The Health of the Prince and Princess," which is drunk with the heartiest of cheers, and though to be sure some of them are rather feeble and chirrupy, of this you may be certain, that whether loud or whispered, they have in them every bit of the strength of those little ones' lungs and voices.

After that the matron remarks that it is getting late and time for little folks to be a-bed, an observation which sobers them somewhat, and the chaplain takes the hint and says few simple words, and then, after they have sung a verse of the old evening hymn, the superintendent winds up the proceedings and the visitors say "Good night" and go.

As Mr. Timberlake and Dr. Rowe descend the stairs they suddenly and with one accord pull out their watches and remark that they have spent four hours in the hospital; then they add that they would never have thought it, and offer apologies which their entertainer will not hear of, and when they have shaken hands in the porch they bid him goodbye, and walk thoughtfully away.

### Talking it Over.

"Ah!" said Mr. Timberlake half to himself, "I have learnt a lesson to-day I shall not be quick to forget. Nervous diseases used to seem very hazy, undefined ailments—something my mind could not grasp—but how terrible, how substantial their reality! Some of the scenes I have witnessed will dwell in my remembrance as long as I live."

"Yes," said Dr. Rowe, "and if only people generally could be persuaded to take the course which old Vinnicombe's bequest has induced you to take—that is to come and see for themselves what the hospitals are doing—the hospital difficulty would be solved. What is it Tennyson says?

'Things seen are greater than things heard.'

And remember that all you have seen is but a sample of the great hospital work Think of the many hospitals you have not visited, think of the machinery never at rest, of the endless round of labours by day and by night, week-day and Sunday. Think of the intrinsic value of the work as it affects the patients and the whole community, of its mercy and compassion, of its economic value, of the unwearying and often self-sacrificing spirit of the workers, and you will form some idea of the most stupendous of all the philanthropic enterprises the world has seen."

### Hospital Sunday.

The little chapel looked as charming on Hospital Sunday as its own inherent beauty and a wealth of exquisite flowers could make it. Boxes and hampers had passed in the day before from far and near, and among them came a splendid gift from Miss Kate Verity. When all had been arranged in such good taste as the chapel-keeper was able to show, the effect was delightful to behold.

Hospital Sunday ranked among the great festivals of the year, and every patient who could be got out of bed with safety—and some who could not—was eager to be present. For not only were they desirous to attend the service—and many of them, poor folk! to add their mites to the offertory—but rumours of the decorations had reached the wards; and when there is not much of colour and beauty in our lives, we value a chance gleam the more.

So some of the poor people who were forbidden by the doctors to leave their beds shed tears over the disappointment, and bewailed their fate to an extent remarkable to witness considering the patience and fortitude usually displayed. And some who came—poor stricken creatures, hailing perhaps from crowded squalid courts and byeways—cried at what they saw; and few who were present remained altogether unaffected until the little service was over.

Mr. Timberlake was in good time—in fact, he arrived as the chapel bell rang, as it always did, full twenty minutes before the hour for the service to begin—for it is no light task to bring together a congregation composed largely of persons unable to walk, while many others are in various degrees of bodily weakness, and move slowly by the aid of sticks, or supported on willing arms.

Having been welcomed by the superintendent, Mr. Timberlake stood watching the assembling of the congregation. And truly it was an interesting sight to see the little groups silently advancing along the corridors, issuing from lifts, coming up and down staircases, and all converging upon the entrance to the chapel. The first comers were in parties of six and eight, each in charge of a nurse or attendant, and constituted the comparatively able-bodied quota from each ward; after them came patients singly, with more palpable effort, but pleased to be able to dispense with help, then poor tremulous forms leaning on the shoulders of the nurses; persons whose legs, as they walked, were jerked this way and that spasmodically; persons with wry necks, persons wearing bandages, persons who shuffled along rather than walked, persons whose every limb and muscle seemed to vibrate, persons with dazed eyes who staggered to and fro, and seemed always giddy; a group of little ones from the children's ward, some limping pitifully, and all trying to act up to nurse's injunctions as to behaviour; several other children carried in arms, then a parade of hospital equipages, pushed by attendants, nurses, and porters; in one of these Jack Russell, who beamed upon Mr. Timberlake as the latter grasped his hand, and in another the poor choreic schoolmaster, looking positively better already—quite a procession, for which every wheel chair in the hospital must have been requisitioned. After these two or three couches on wheels, each with its occupant, and finally a bevy of nurses and probationers, looking bright and comely in their dresses of grey or pink, according to grade, the sisters graceful in dark blue serge and high white caps and white aprons—snowy enough to offer a mightily good testimonial to London laundry work; attendants, male nurses and porters, smart in dark uniforms, a galaxy of buxom women servants, and a small sprinkling of visitors.

When Mr. Timberlake took the seat reserved for him in the corner allotted to the several resident officials he found that everybody had been set down in the place assigned to him with a precision and absence of disorder which comes of long practice, and though there was not a seat to spare, and both the superintendent and matron confessed to a little anxiety as regards the gangways in case a fit should occur among the epileptic or hysterical patients, as a matter of fact nothing whatever took place to interrupt the proceedings.

Being Hospital Sunday, the chaplain had obtained the help of a brother clergyman, and two or three friends with good voices had come in to strengthen the singing, but the services of the latter were scarcely needed, for the congregation was always encouraged to join in heartily, and the tunes chosen were familiar and soul-stirring.

A Short Service.

The service was a much abbreviated version of the "Order for Morning Prayer," and Mr. Timberlake said afterwards he never remembered to have felt so completely the power [illegible] religion as when he looked about on this congregation [illegible] suffering people reverently intent upon their devotions, and remembered that to many of them cessation of pain would be in itself an earthly heaven, and that many more might have said in complaint rather than submission, "Thou hast afflicted me from my youth up."

Yet there they were, singing hymns of thankfulness and hope, neither murmuring at their afflictions nor questioning the divine truths set forth before them, nor refusing the proffered consolation.

What came of it.

When all had dispersed, Mr. Timberlake and the superintendent paid a visit to the chaplain, in the latter's room, and had a friendly chat. What was said need not be recorded, but we know this, that the amount the chaplain was enabled to hand over to the Hospital Sunday Fund—and not a penny of it was old Vinnicombe's money—was so large that the secretary of the fund has not yet ceased to wonder where it all came from; and one social economist of great dialectical skill has convinced himself that if the hospital in question receives patients who can afford to give away money like that, why it certainly ought not to share in the division of the fund, which was clearly meant to be devoted to the poor.

Curiously enough, other hospital chaplains paid in sums much larger than usual, and although the secretary has never yet received an explanation, we may mention that it was owing to the campaign upon which Mr. Timberlake had entered the day before among his old friends in the City; for he persisted so long and so loudly, that one and another was persuaded at last to attend the service at this and that hospital, and to carry with them in each case a substantial cheque. It is simply amazing what power for good men have with their fellows if they will only exert it; and the merchant princes of London—the most generous men in the world if you only know how to enlist them in a good cause, which few of us do, more's the pity—agreed among themselves that what they admired about old Timberlake was that "whatever he took up, you might be quite sure he would go right through with it."

---

The last part of this narrative is the only portion which can be rightly considered apocryphal. An opportunity shortly offers (Sunday, June 10th, 1888) to bring this incident into complete harmony with the remainder.

B. Burford Rawlings.

